# Stress Profile in Remotely Piloted Aircraft Crewmembers During 2 h Operating Mission

**DOI:** 10.3389/fphys.2018.00461

**Published:** 2018-05-07

**Authors:** Anna Valenzano, Fiorenzo Moscatelli, Antonietta Messina, Vincenzo Monda, Raffaele Orsitto, Giovanna Zezza, Giovanni Fiorentino, Monica Salerno, Antonio I. Triggiani, Andrea Viggiano, Maria P. Mollica, Marco Carotenuto, Marcellino Monda, Giuseppe Cibelli, Giovanni Messina

**Affiliations:** ^1^Department of Clinical and Experimental Medicine, University of Foggia, Foggia, Italy; ^2^Department of Experimental Medicine, Section of Human Physiology and Unit of Dietetic and Sport Medicine, Università degli Studi della Campania Luigi Vanvitelli, Naples, Italy; ^3^32nd Wing, Italian Air Force, Amendola, Italy; ^4^Department of Medicine, Surgery and Dentistry “Scuola Medica Salernitana”, University of Salerno, Fisciano, Italy; ^5^Department of Biology, Università degli Studi di Napoli Federico II, Naples, Italy; ^6^Clinic of Child and Adolescent Neuropsychiatry, Department of Mental Health, Physical and Preventive Medicine, Università degli Studi della Campania Luigi Vanvitelli, Naples, Italy

**Keywords:** drone, military, galvanic skin response, α-amylase, stress

## Abstract

Emotional stability plays a key role in individual and team performance during both routine activities and management of unexpected emergencies. Using a psycho-physiological approach, the stress response was investigated in drone operators in service.

**Methods:** Salivary α-amylase (sAA), galvanic skin response (GSR) and anxiety were assessed over a 2-h operating flight.

**Results:** Compared to baseline values, GSR and sAA values increased in operating conditions. Moreover, these values were higher in Pilots than in Sensor Operators, indicating that their stress response was greater. These results were associated with an increase in anxiety level, highlighting a relationship between autonomic reactivity and anxiety.

**Conclusion:** This is the first report providing experimental evidences of the stress response related to Remotely Piloted Aircraft operations.

## Introduction

Remotely Piloted Aircraft (RPA), commonly called “drone,” is a flying vehicle without a human pilot that represent an innovative war fighting technology. New advancements in satellite communication systems and in aviation technologies, allow bases to provide around-the-clock support, 7 days a week, to military operations across the globe (Chappelle W. et al., 2014). Recently, the use of drones for surveillance and reconnaissance during air support missions, have increased. Long-working hours, ergonomic design of the workstation, continuous processing of auditory and visual data during flight missions, and sustaining vigilance, represent potential RPA-related operating stressors (Goldstein and Kopin, 2008; Tvaryanas and MacPherson, 2009), with possible negative consequences for the health and well-being of RPA operators (Carretta, 2013). Unfortunately, there is still a paucity of studies about drone operators, due to the restricted access to them (Chappelle W. et al., 2014). Emotional stability plays a key role in individual and team performance during routine activities and during management of unexpected emergencies (Phitayakorn et al., 2015), and it can be assessed by physiologic and psychologic measurements (Bradley and Lang, 2002). Physiologic measurement of emotional stability includes several kinds of devices conceived to record activation of the hypothalamic–pituitary–adrenal axis through the elevation of heart rate, respiration rate, blood pressure, blink rate, and pupil dilatation (Timmermans et al., 2013). A considerable amount of research studies identified the: hypothalamic-pituitary-adrenal (HPA) axis, the sympathoneural (SN) systems, and the sympathetic-adreno-medullary (SAM) system, as the main components of the psychobiological response to stress. Activation of these systems resulting in both the secretion of glucocorticoids and catecholamines into the blood stream (Lundberg and Frankenhaeuser, 1980; Chrousos and Gold, 1992; Peters et al., 1998; Young et al., 2005; Messina et al., 2013). Even though the physiological interconnection between the systems above is well-proven, their reaction to stress is different. Neuroendocrine and autonomic responses are related to type and intensity of the stressors, and are strictly dependent on the individual’s experience (Goldstein and Kopin, 2008; Tvaryanas and MacPherson, 2009).

Individual response to stressors can be assessed by a non-invasive method such as Galvanic skin response (GSR) and salivary α-amylase (sAA).

The GSR has long been considered as a method to reveal physiological and mental stress (Tvaryanas et al., 2006; Chappelle W.L. et al., 2014), because it is a well-known validated marker of sympathetic activity (Sequeira et al., 2009). The level of sAA is also considered as an effective measure of stress, since it resembles circulating catecholamines (Chatterton et al., 1996).

Using hormonal regulation as a measure of stress is problematic to researchers, since changes in serum norepinephrine levels occur with a delay of 20–30 min in response to loading stress. Conversely, stimulation of salivary amylase secretion by direct innervations, results in an increase within few minutes, considerably quicker than the hormonal regulation induced response (Skosnik et al., 2000). The salivary glands act as an amplifier to the low level of norepinephrine, reacting to the psychological stressors faster than blood cortisol level. Therefore, sAA assay was found to be a good index of psychological stress (Chatterton et al., 1996, 1997).

Another aspect to consider regarding military pilots and their crew members is Post-traumatic stress disorder (PTSD). PTSD has been increasingly discussed in the media in recent years following the homecoming of the British soldiers serving in Iraq and Afghanistan (Pitman et al., 2012; King and Smith, 2016). Many are returning with debilitating conditions. Between January 1st 2006 and 31st December 2014, 2188 UK military and civilian personnel were admitted to UK field hospitals and categorised as wounded in action (MOD, 2015). The International Statistical Classification of Diseases and Related Health Problems (ICD-10) (Murrison, 2010) classifies PTSD as a disorder which emerges as a delayed or protracted response to an experience or situation that is expected to cause substantial distress to almost anyone due to the extreme threatening or catastrophic nature of the event. The disorder is characterized by a continuing sense of numbness and emotional blunting in which the sufferer detaches themselves from relationships and becomes unresponsive to surroundings. Typical features include repeated experiences of reliving the traumatic experiences in disturbing memories (flashbacks), distressing dreams or nightmares, and a state of hyper-arousal with hyper-vigilance. Anhedonia is frequent and comorbid conditions of anxiety and depression are common, also suicidal ideation is not rare. The onset of PTSD follows the traumatic event, arising from a few weeks to months after (World Health Organization [WHO], 2016).

In order to assess the levels of stress and workload experienced by RPA operators (Tvaryanas and MacPherson, 2009; Chappelle W. et al., 2014), sAA assay and GSR have the potential to provide researchers with tools for an objective measurement of stress during operating conditions (Chatterton et al., 1996, 1997).

This study is aimed at investigating the stress response in twelve experienced adult male crewmembers (six Pilots and six Sensor Operators) of MQ-9 Predator B, in daytime operating conditions, using a psycho-physiological approach.

## Materials and Methods

### Subjects

Twelve experienced adult male crewmembers participated voluntarily to this study. The cohort of participants included: six Pilots, age 31.6 ± 2.2, body mass 75.8 ± 4.9 kg, height 166.1 ± 2.8 cm; and six Sensor Operators, age 39.2 ± 6.7, body mass 73.9 ± 5.2 kg, height 176.1 ± 5.1 cm, all assigned to the 28° RPA Squadron, based at 32nd Wing in Amendola (Italy). There are no statistically significant differences in age, body mass, and height between pilots and sensor operators. All the recruited subjects underwent a clinical assessment to ascertain the absence of cardiovascular disease and endocrine disorders. Furthermore, they were not taking any kind of medication. The participants were asked to abstain from physical activity for 2 days before the test. Each participant was informed about the purpose of the study and they signed informed consent, according to the Declaration of Helsinki. The study was approved by Institutional Ethics Committee of the University of Foggia.

### Study Protocol

Subjects were instructed to normally eat and drink and to sleep for at least 8 h the night before the test. Salivary samples and GSR were assessed during a 2-h daytime of operating flight, scheduled from 10:00 to 12:00 a.m. Prior to starting and after completing their operating program, the participants completed the State-Trait Anxiety Inventory (STAI-Y1) questionnaire (Buonanno et al., 2017).

### Hormonal Assay

Salivary samples were collected using cotton swabs (Salivette, Sarstedt, Rommelsdorf, Germany), before operating flight (T0: baseline condition), every 30 min during operating flight (30 min-60 min-90 min-120 min) and 15 and 30 min after the operating flight (15 min post and 30 min post). Participants had to keep the cottons swab into the mouth and chewing it for at least 2 min. Then, they had to put it into a sterile plastic tube. Samples were returned within 4 h to the laboratory and stored at -20°C until the sAA assay could be performed. A salivary kit (Salimetrics LLC, State College, PA, United States) was used to check for the absence of blood contamination. Salivary samples were centrifuged at 1500 ×*g* for 15 min at 4°C. Fifty microliters of saliva were used for duplicate analysis. All analyses were performed using the same assay-plate. Salivary concentrations were processed using a commercial salivary kit (Salimetrics LLC, State College, PA, United States) and revealed using a standard plate reader (PowerWave XS, Bio-Tek Instruments, United States) with a 450 nm filter. Intra- and inter-assay coefficients of variation were 4.2 and 7.8%, respectively.

### GSR Measurement

Galvanic skin response measurement involves capturing the autonomic nerve response in terms of the activity of sweat glands, it measures the electrical resistance of the skin. As stress levels increase, changes in the electrical resistance of the skin are detected by GSR sensors. It is also known as the electrodermal response, psychogalvanic reflex, or skin conductance response.

All GSR parameters were recorded simultaneously with a Holter device (SenseWear Pro_2_ armband, BodyMedia, Inc., Pittsburgh, PA, United States) placed on the right arm at the mid point between the acromion process and olecran on process, following manufacturer recommendation.

The armband is worn on the back of the upper arm, which enables continuous physiological data collection outside a laboratory environment. Using metallic sensors close to the skin, the armband collects biorhythmic data in real time, with a configurable sample rate, and gathers raw physiological data such as GSR. The GSR data were acquired within 30-min intervals, following saliva collection.

### Statistical Analysis

The R Project software (version 3.3.1) was used for statistical analyses. Normality of distributions was checked using the Shapiro–Wilk test: the variables that did not pass the check, were log-transformed and checked again. Analysis of variance (ANOVA) for repeated measures was performed to investigate GSR and sAA changes over time in all subject, using Tukey’s “Honest Significant Difference” (HSD) for *post hoc* multiple comparisons. Two way mixed ANOVA, with one within subjects factor and one between groups factor was used. Time was the within subjects factor; Group (sensor/pilot) was the between subjects factor. The variables that passed the Mauchley Sphericity test were tested using the Greenhouse-Geisser test or the Huynh–Feldt test.

Paired *T*-test was performed to investigate the differences in self-reported anxiety between pre- and post-operating flight. Statistical significance was determined using *p* ≤ 0.05. Pearson correlation were performed in order to investigate the percentage increase between STAI (Pre/post) and GSR (Pre/15 post), and between STAI (Pre/post) and sAA (Pre/15 post).

## Results

**FIGURE 1:**
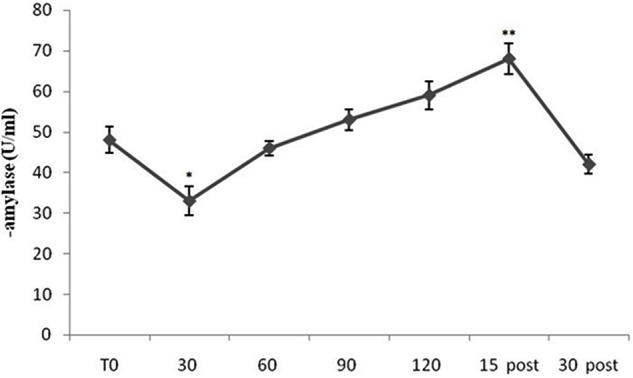
Salivary α-amylase (sAA) levels from all subjects (both Pilots and Sensors Operators) over time. sAA levels were measured before operating flight (T0: baseline condition), every 30 min during operating flight (30 min-60 min-90 min-120 min), and 15 and 30 min after the operating flight (15 min post and 30 min post). ^∗^*p* < 0.05; ^∗∗^*p* < 0.01.

**Figure 1** shows mean sAA levels from all subjects (both Pilots and Sensors Operators) over time. sAA values increased during the flight period, compared to baseline, with peak values at 15-min after the completion of the flight. The ANOVA showed a significant difference in sAA levels [*F*(10,60) = 15.69; *p* < 0.001]. The *post hoc* test showed significant differences between T0 and 30 min (*p* < 0.05), and between T0 and 15 min post (*p* < 0.01). Interestingly, in the early stages of flight, sAA levels were lower than baseline.

**FIGURE 2:**
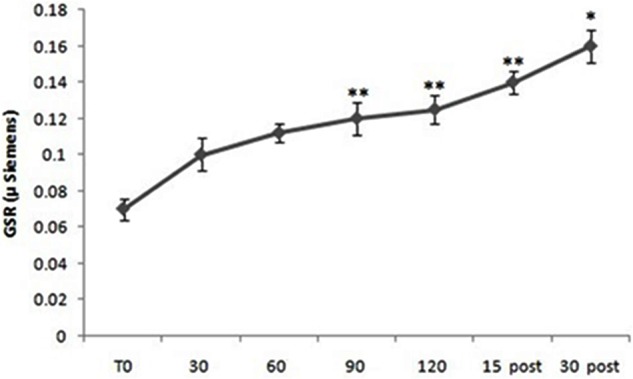
Galvanic skin response (GSR) levels from all subjects (both Pilots and Sensors Operators) over time. GSR levels were measured before operating flight (T0: baseline condition), every 30 min during operating flight (30 min-60 min-90 min-120 min), and 15 and 30 min after the operating flight (15 min post and 30 min post). ^∗^*p* < 0.05; ^∗∗^*p* < 0.01.

Repeated measure ANOVA showed significant differences in GSR levels considering all subjects [*F*(10,60) = 3.88; *p* < 0.01]. *Post hoc* comparison showed significant differences between T0 and 60 min (*p* < 0.01), between T0 and 90 min (*p* < 0.01), between T0 and 120 min (*p* < 0.001), between T0 and 15 min post (*p* < 0.01) and between T0 and 30 min post (*p* < 0.05) (**Figure 2**).

**FIGURE 3:**
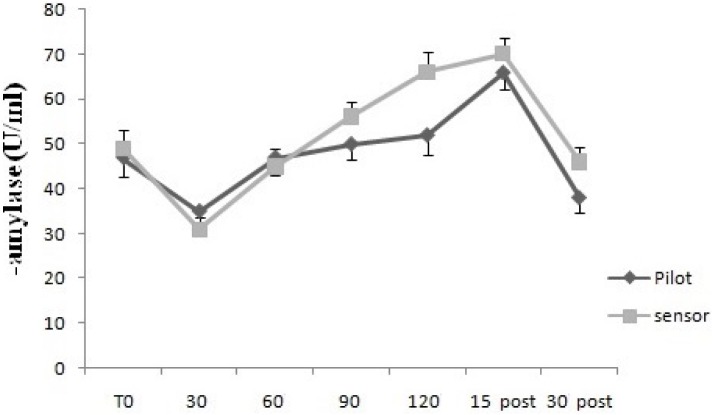
Galvanic skin response levels over time for Pilots and Sensor Operators. GSR levels were measured before operating flight (T0: baseline condition), every 30 min during operating flight (30 min-60 min-90 min-120 min), and 15 and 30 min after the operating flight (15 min post and 30 min post).

**FIGURE 4:**
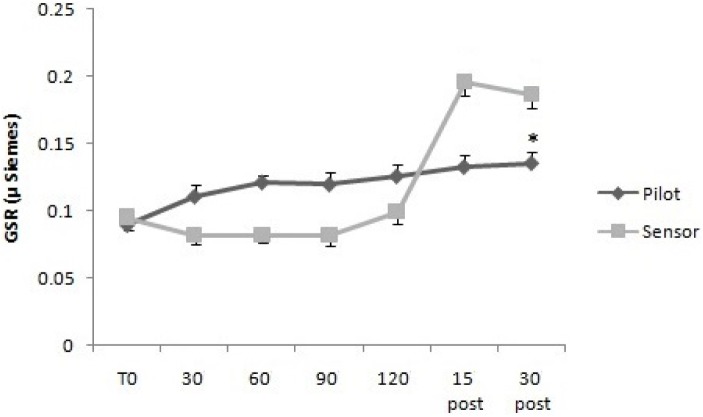
Salivary α-amylase levels over time for Pilots and Sensor Operators. sAA levels were measured before operating flight (T0: baseline condition), every 30 min during operating flight (30 min-60 min-90 min-120 min), and 15 and 30 min after the operating flight (15 min post and 30 min post). ^∗^*p* < 0.05.

For GSR, the two way mixed ANOVA model showed that there was not statistical significant main effect of Time, neither an interaction between Time and Group, using Huynh–Feldt test, since the hypothesis of sphericity was violated. For sAA, the two way mixed ANOVA model showed a statistical significant main effect of Time [*F*(2.63,26.3) = 7.95, *p* < 0.01], but not an interaction between Time and Group, since on using Huynh–Feldt test, the hypothesis of sphericity was violated (**Figures 3, 4**).

The comparison of self-reported anxiety ratings between pre- and post-flight was statistically significant considering all participants grouped (Pre 27.3 ± 6.5, Post 31.7 ± 5.8; *p* < 0.01), but no significant differences emerged between the two experimental groups.

Significant correlation in percentage increase emerged between STAI (Pre/post) and GSR (Pre/15 post) (*r* = 0.21; *p* < 0.05), and between STAI (Pre/post) and sAA (Pre/15 post) (*r* = 0.29; *p* < 0.01).

## Discussion

To our knowledge, this is the first research study examining both autonomic responses and the anxiety associated with RPA operating. Our results demonstrated that during a 2-h daytime of RPA operating, GSR and sAA significantly increased compared to basal values both in Pilots and in Sensor Operators. This demonstrated an augmented sympathetic-adrenal response occurring during the task.

These results are consistent with previous findings in parachutists (Messina et al., 2015). The authors studied physiological reactions and emotional response. They presented data on the relationship between hormonal, autonomic and both somatic and cognitive anxiety in subjects practicing sport parachuting. They reported that Somatic Anxiety is correlated to the sympatho-neural systems and the sympathetic- adreno-medullary activation, and that coordinated regulatory mechanisms for endocrine and autonomic responses to stressor may exist. These results confirmed that GSR has the potential to provide researchers with a tool for objectively measuring stress during operational conditions.

An important aspect of this study concerns the different behavior in the operators’ stress response. During stress, homeostatic adaptive processes are activated to induce behavioral and physiological changes (Bali and Jaggi, 2015). We found that during the task, both GSR and sAA values were higher in Sensor Operators than in Pilots, suggesting that the level of stress experienced by the Sensor Operators was higher. We hypothesize that this depends on both operating tasks, and environmental situations related to RPA. Recent studies concerning the psychobiology of stress benefited from a multivariable model. sAA as a surrogate biomarker of the autonomic nervous system (ANS) was found to improve such a model, defining a better picture of the potential dysfunction of stress response in different subjects (Glenn et al., 2015). Therefore, sAA has revealed to be a good non-invasive biomarker for stress-related changes reflecting the activity of the sympathetic branch of the ANS. Further proof of the influence of situational factors in conditioning the stress response in the crewmembers was provided by the general trend of sAA values during the daytime flight. In fact, we may assume that sAA baseline levels were high when anticipation levels were highest (before the taking off) (Chieffi et al., 2017).

It has been reported that the secretion of sAA is regulated by norepinephrine release in the salivary glands (Yamaguchi et al., 2004; Nater et al., 2005; Granger et al., 2007; Perroni et al., 2009). Accordingly, sAA level increases in presence of stressors, which, in turn increases plasma catecholamines (Chatterton et al., 1997; Skosnik et al., 2000; Capranica et al., 2012; Messina et al., 2016, 2017). The level of sAA has been successfully used to test the impact of social and competitive stress, such as performance in front of an audience (Chatterton et al., 1996; Rohleder et al., 2004; Gordis et al., 2006), testing (Yamaguchi et al., 2004), competition (Kivlighan and Granger, 2006; Chiodo et al., 2011) and physical stress (Chatterton et al., 1997; Wetherell et al., 2006; Perroni et al., 2010).

In this study the level of sAA decreases after 15 min from the beginning of the activity. This decline is probably due to a phase of decreased stress. In fact the first period of the misson turns out to be very strenuous due to the take-off operations of the airplane. Furthermore, high levels of sAA are also found in the recovery phase after flight mission. This increase is due to the proximity of another stressful period that corresponds with the landing phase of the plane.

Psychological stress has an influence on several psychological processes in healthy subjects and in people who suffer from psychiatric disorders (Swaab et al., 2005; Wolf, 2009; Foley and Kirschbaum, 2010; Giles et al., 2014). Stress is thought to influence mood (Van Eck et al., 1996; Lieberman et al., 2002), memory (Roozendaal et al., 2009), and decision-making (Starcke et al., 2011; Moscatelli et al., 2016; Villano et al., 2017). Furthermore, acute psychological stress activates the HPA axis and sympathetic nervous system (SNS), producing higher levels sAA (Monda and Pittman, 1993; Monda et al., 1994; Giles et al., 2014).

In order to comprehend the influence of stress on physical and cognitive performance, it is fundamental to understand the different manifestations of the anxiety. Increases in sAA concentration and GSR were found to be associated with increases in somatic anxiety. This positive relationship might support the hypothesis that arousal would result in increases in GSR and sAA levels. Our results showed a moderate level of anxiety in RPA operators. This is likely related to increased arousal and perceived self-control, and might facilitate an increase in performance and self-confidence (Julian, 2011). According to our data, changes in sAA levels and GSR may be interpreted as a “situational” stress and arousal.

Overall, considering together the physiological reactions and emotional response, this is the first data examining the relationship between autonomic responses and anxiety in RPA operators. In conclusions, corroborating previously published data, these results suggest that anxiety is related to sympathetic adreno-medullary activation (Messina et al., 2015).

## Author Contributions

AVa, FM, AM, VM, and GM: conceived the study, participated in its design, and wrote the manuscript. RO, GZ, GF, MS, AT, MPM, and MC: contributed to the conception and design. AVa, MM, GC, and GM: drafted the article and revised it critically for important intellectual content. GM: final approval of the version to be published. All authors read and approved the final manuscript.

## Conflict of Interest Statement

The authors declare that the research was conducted in the absence of any commercial or financial relationships that could be construed as a potential conflict of interest.
